# Standardisation of conventional and advanced iterative reconstruction methods for Gallium-68 multi-centre PET-CT trials

**DOI:** 10.1186/s40658-021-00400-8

**Published:** 2021-07-17

**Authors:** Georgios Krokos, Lucy C. Pike, Gary J. R. Cook, Paul K. Marsden

**Affiliations:** 1 Department of Biomedical Engineering, School of Biomedical Engineering and Imaging Sciences, King’s College London, London, UK; 2 Department of Cancer Imaging, School of Biomedical Engineering and Imaging Sciences, King’s College London, London, UK

**Keywords:** Standardisation, PET-CT, Gallium-68, Image reconstruction

## Abstract

**Purpose:**

To assess the applicability of the Fluorine-18 performance specifications defined by EANM Research Ltd (EARL), in Gallium-68 multi-centre PET-CT trials using conventional (ordered subset expectation maximisation, OSEM) and advanced iterative reconstructions which include the systems’ point spread function (PSF) and a Bayesian penalised likelihood algorithm (BPL) commercially known as Q.CLEAR. The possibility of standardising the two advanced reconstruction methods was examined.

**Methods:**

The NEMA image quality phantom was filled with Gallium-68 and scanned on a GE PET-CT system. PSF and BPL with varying post-reconstruction Gaussian filter width (2–6.4 mm) and penalisation factor (200–1200), respectively, were applied. The average peak-to-valley ratio from six profiles across each sphere was estimated to inspect any edge artefacts. Image noise was assessed using background variability and image roughness. Six GE and Siemens PET-CT scanners provided Gallium-68 images of the NEMA phantom using both conventional and advanced reconstructions from which the maximum, mean and peak recoveries were drawn. Fourteen patients underwent ^68^Ga-PSMA PET-CT imaging. BPL (200-1200) reconstructions of the data were compared against PSF smoothed with a 6.4-mm Gaussian filter.

**Results:**

A Gaussian filter width of approximately 6 mm for PSF and a penalisation factor of 800 for BPL were needed to suppress the edge artefacts. In addition, those reconstructions provided the closest agreement between the two advanced iterative reconstructions and low noise levels with the background variability and the image roughness being lower than 7.5% and 11.5%, respectively. The recoveries for all methods generally performed at the lower limits of the EARL specifications, especially for the 13- and 10-mm spheres for which up to 27% (conventional) and 41% (advanced reconstructions) lower limits are suggested. The lesion standardised uptake values from the clinical data were significantly different between BPL and PSF smoothed with a Gaussian filter of 6.4 mm wide for all penalisation factors except for 800 and 1000.

**Conclusion:**

It is possible to standardise the advanced reconstruction methods with the reconstruction parameters being also sufficient for minimising the edge artefacts and noise in the images. For both conventional and advanced reconstructions, Gallium-68 specific recovery coefficient limits were required, especially for the smallest phantom spheres.

**Supplementary Information:**

The online version contains supplementary material available at 10.1186/s40658-021-00400-8.

## Introduction

Standardisation is a key aspect when conducting PET multi-centre clinical trials in order to minimise the sources of variation between participating sites. Moreover, it improves the robustness of the performed analyses, especially when quantification is of importance. Unaccounted-for inter-scanner variations could lead to significant differences in image noise and the accuracy of standardised uptake values (SUVs) which could in turn significantly reduce the power of the conducted studies [1, 2].

The importance of standardisation has been acknowledged by the scientific community especially in trials using ^18^F-fluoro-2-deoxy-d-glucose (FDG). This is mainly due to the radiotracer’s well-established role in oncology and its availability in most PET centres, making it logistically easier to conduct multi-centre trials. Gallium-68-based radiotracers are increasingly finding their way into clinical PET applications owing to the introduction of Germanium-68/Gallium-68 generators, their fast target localisation, their fast blood clearance and the short physical half-life of Gallium-68 (T_1/2_ = 68 min) [3]. The most commonly used, ^68^Ga-prostate specific-membrane antigen (PSMA), has exhibited high specificity in prostate cancer with high proportion of changes in management (21%) of patients undergoing the scan [4, 5]. Similarly, ^68^Ga-tetraazacyclododecanetetraacetic acid-DPhe1-Tyr3-octreotate (DOTATATE) has shown advantages in the management of patients, especially in well-differentiated neuroendocrine tumours compared to ^18^F-FDG [6]. Published guidelines for scanning protocols with those tracers recommend an injected activity of up to 200MBq and acquisition time of 2–4 min per bed position [7, 8]. Taking also into account the distinctly different physical characteristics of the two radionuclides (approximately 5 times longer positron range, with approximate mean ranges of 0.6 mm for Fluorine-18 and 2.9 mm for Gallium-68 in water [9], half the physical half-life and 8% lower positron yield for Gallium-68 compared to Fluorine-18), it becomes evident that the Fluorine-18 based guidelines from EANM Research Ltd. (EARL) might not be directly applicable for [68Ga]-labelled tracers [10]. Huizing et al. reported greater variation between sites for Gallium-68 and an 11% decrease in recovery coefficient compared to Fluorine-18 when scanning the NEMA Image Quality phantom as per the EARL guidelines [11]. In their study, the main source of difference between the two tracers was due to inaccuracies in the scaling factor for Gallium-68 in the dose calibrators as set by the manufacturers and was then partly alleviated by scaling the recovery coefficients by the measured discrepancy [12]. Soderlund et al. also reported an inverse relationship between recovery and positron range when comparing phantom acquisitions reconstructed with the same parameters, indicating the need for tracer specific limits [13].

Incorporation of point spread function (PSF) in iterative reconstruction is reported to lead to images with significantly higher contrast compared to standard iterative algorithms, especially for small lesions, but resulting in the appearance of edge artefacts in lesions [14, 15]. PSF reconstruction is now offered by most PET-CT systems. As in non-PSF iterative reconstruction, three of the main user defined parameters on the scanner are the number of subsets, number of iterations and the post-reconstruction Gaussian filter. The iterations and the subsets define the agreement between measured and estimated projection data and their product (effective iterations) is usually large enough (typically between 32 and 48) to ensure convergence of the radioactivity concentration in the region of interest. However, the improvement in accuracy by increasing the number of effective iterations comes at the cost of increased noise, which adds uncertainty in identifying small lesions. The most commonly used method for suppressing this noise is the application of a post-reconstruction Gaussian filter which performs a weighted averaging in neighbouring pixels and is typically 3–10 mm wide. The downside of this approach is the blurring caused on the edges of regions and apparent decreased uptake in metabolically active lesions which could potentially counter the benefit of using PSF reconstruction in the first place. Consequently, there is no consensus in the literature on the input values that should be applied, especially for the width of Gaussian filter, which could lead to significant variations in SUVs [16, 17].

GE Healthcare has recently further expanded on the PSF reconstruction algorithm by including a Bayesian penalised likelihood (BPL) reconstruction method (Q.CLEAR), which is claimed to increase image accuracy as it iterates the image until full convergence has been achieved. The only input parameter is the penalisation factor (β) which regulates the level of noise in the images [18]. Recent qualitative studies using Fluorine-18 labelled tracers in clinical data [19–21], reported an optimum value of β=300–400. Quantitative assessments [19, 21] also revealed that this value provided a higher signal-to-noise ratio when compared to standard iterative reconstruction. For ^68^Ga-PSMA clinical scans, ter Voert et al. reported an optimum SNR for β values between 400 and 550 [22]. Effects such as edge artefacts and effect of noise and contrast recovery in regions of different sizes can be difficult to assess qualitatively and, to our knowledge, a quantitative analysis on phantom data for the investigation of the optimum β value for Gallium-68 labelled tracers still needs to be conducted. Moreover, as Q.CLEAR and PSF reconstructions become the standard of care for many sites, standardisation procedures for Gallium-68 multi-centre trials need to be established when using those methods.

In this study, we initially investigated the optimum level of Gaussian filtering for traditional PSF reconstruction and the optimum penalisation factor for Q.CLEAR in terms of edge artefact suppression. The applicability of the EARL Fluorine-18 defined accreditation limits for the standardisation of six UK-based scanners planning to participate in Gallium-68 multi-centre trials was reviewed for the most commonly used ordered subset expectation maximisation reconstruction (OSEM) while standardisation of the advanced reconstruction techniques was also attempted based both on their recovery coefficients and noise characteristics. As it can be argued that the NEMA phantom might be an unrealistic approach for evaluating the differences between two reconstruction methods in real clinical data with lesions of various metabolic activities, sizes and neighbouring tissues, the agreement between PSF and Q.CLEAR reconstruction was also evaluated using clinical scans of patients scanned with ^68^Ga-PSMA.

## Methods

### Phantom data

#### Optimisation of advanced reconstruction methods

For the investigation of the performance of Q.CLEAR and its harmonisation with PSF reconstruction, the NEMA IEC PET Body Phantom™ (Data Spectrum) was scanned on the GE Discovery 710 PET-CT scanner. Gallium-68 was inserted in all six spheres (37, 28, 22, 17, 13, and 10 mm) and the background region, achieving radioactivity concentrations of 19.9 kBq/ml and 2.4 kBq/ml, respectively. The lung insert packed with styrofoam beads was also included in the background. The raw data were reconstructed with Q.CLEAR with varying β values (200, 400, 600, 800, 1000 and 1200) and with PSF using 2 iterations and 24 subsets and varying Gaussian post-reconstruction filter width (2, 4, 5 and 6.4 mm). Both methods use the OSEM algorithm, including time of flight (TOF) and PSF but with the addition of a BPL method in Q.CLEAR. A straightforward nomenclature to highlight the main differences of the two reconstructions would be OSEM+TOF+PSF_w_ with w being the filter width in mm for the traditional PSF reconstruction and OSEM+TOF+PSF+BPL_β_ with β being the penalisation factor for the Q.CLEAR method. For better legibility though, the two methods will be referred to simply as PSF_w_ and BPL_β_. The figures for all analyses were generated using Matlab R2020b.

#### Multi-centre Fluorine-18 and Gallium-68 acquisition

All scanners included in this study had been accredited by the UK PET Core Lab (http://www.ncri-pet.org.uk/) for participation in Fluorine-18-based PET-CT multi-centre trials. For the accreditation process, the same specification limits as defined by EARL standard 1 were used.

The NEMA IEC PET Body Phantom™ (Data Spectrum) was scanned on 12 Siemens and GE scanners with Fluorine-18. Local sites were provided with a standardised filling procedure and asked to acquire the phantom using local clinical protocols for FDG and a sphere-to-background ratio of 4.8:1 to approximate the average tumour-to-background ratio as observed in clinical FDG studies [23, 24]. A summary of the scanner models and reconstruction parameters are shown in Table 1. The sphere-to-background ratio was 4.61 ± 0.42 (average ± standard deviation) with all six spheres containing radioactivity and the lung insert included in the background region. All images were reconstructed both with OSEM+TOF_w_ and PSF_w_. The number of effective iterations was maintained the same between OSEM+TOF_w_ and PSF_w_ as it has been previously shown that the convergence of the recovery coefficient is similar between the two for that level of post-reconstruction smoothing [25].

**Table 1:** Reconstruction and acquisition parameters for all NEMA IQ scans using Fluorine-18 and Gallium-68

No of scanners	Scanner	Iterations and subsets	Gaussian Filtering (mm)	Voxel dimensions (mm^**3**^)	Time/bed position or scan speed
**Fluorine-18 scans**					
1	Siemens mCT Flow	2i21s	5	4.1x4.1x3.0	0.8 mm/s^a^
2	Siemens mCT Flow	2i21s	5	4.1x4.1x3.0	1.1 mm/s^b^
3	Siemens mCT Flow	2i21s	5	4.1x4.1x2.0	3 min/bed position
1	Siemens mCT Edge	2i21s	6	4.1x4.1x3.0	3 min/bed position
1	GE Discovery MI	3i17s	5	2.7x2.7x2.8	3 min/bed position
2	GE Discovery 690	2i24s	6.4	2.7x2.7x3.3	3 min/bed position
2	GE Discovery 710	2i24s	6.4	2.7x2.7x3.3	3 min/bed position
**Gallium-68 scans**					
1	GE Discovery MIDR	3i16s	6.4	2.7x2.7x3.3	5min/bed position
1	GE Discovery 690	2i24s	6.4	5.5x5.5x3.3	4 min/bed position
1	Siemens mCT Flow	2i21s	5	4.1x4.1x2.0	4 min/bed position
1	Siemens TrueV	2i21s	5	4.1x4.1x5.0	3 min/bed position
1	GE Discovery 710	2i24s	6.4	2.7x2.7x3.3	4 min/bed position
1	GE Discovery 710	2i24s	6.4	2.7x2.7x3.3	3 min/bed position

^a^Corresponds to 2.5 mm/bed position according to Siemens instructions for protocol definition

^b^Corresponds to 2 mm/bed position according to Siemens instructions for protocol definition

Two Siemens and four GE scanners provided acquisitions of the NEMA IQ phantom with Gallium-68 again using centrally distributed procedures and with 8.22 ± 0.53 sphere-to-background ratio. The higher sphere-to-background ratio compared to the FDG scans was selected in order to take into account the higher tumour-to-background ratio observed in ^68^Ga-PSMA scans. A conservative approach was selected though compared to the published contrast in clinical images, which also enables the results from this study to be compared with the existing literature [16, 23, 26, 27]. The reconstruction parameters were based on the local clinical protocols for whole-body ^68^Ga-PSMA scans. One Siemens scanner (Siemens mCT Flow) provided both OSEM+TOF_5_ and PSF_5_ reconstructions but for the second one (Siemens TrueV), in which these options were not available, only the OSEM_5_ reconstruction was applied. All GE scanners reconstructed the data using OSEM+TOF_6.4_. Three scanners (two Discovery 710 and Discovery 690) also provided a PSF_6.4_ reconstruction and three sites (two Discovery 710 and Discovery MIDR) a BPL_800_ reconstruction.

#### Analysis of phantom data

All sphere regions were semi-automatically drawn using a 3D isocontour at 50% of the maximum value within each sphere [10, 16] using MIM Software Inc. 6.9.3. Also, six concentric, circular regions were drawn in the background with diameters equal to the nominal diameter of the hot sphere regions. Those were replicated 12 times and on five different slices (360 in total) as described by Tong et al. [25]. To assess the compliance of the spheres with the EARL specifications (http://earl.eanm.org/ accessed on January 22, 2021), the recovery coefficient RC was used:

1 $$ RC=\frac{C_m}{C_{dc}} $$

C_m_ is the measured radioactivity concentration in the images and C_dc_ is the radioactivity concentration inserted in the spheres as measured from the radionuclide calibrator, corrected for decay and residual. For the sphere regions, the RC was measured for the voxel with the maximum reconstructed activity concentration (RC_max_), for the mean activity (RC_mean_) and for a small (1cm^3^) spherical region of interest in the area of the highest reconstructed activity concentration (RC_peak_) which was allowed to extend outside the boundaries of the delineated sphere region [28].

For comparison between scanners and acquisitions, the contrast recovery coefficient CRC was used in order to account for variations between sphere-to-background ratios and investigate the effect of varying region size [29].

2 $$ CRC=\frac{C_m-{C}_{bkg.m}}{C_{dc}-{C}_{bkg. dc}} $$

C_bkg.m_ is the measured radioactivity concentration in the background from the images and C_bkg.dc_ the inserted radioactivity concentration in the background as measured from the radionuclide calibrator, corrected for decay and residual. The CRC_max_, CRC_mean_ and CRC_peak_ were calculated.

The noise in the images was assessed by measuring background variability (BV) and image roughness (IR) [25]:

3 $$ {BV}_r=\frac{\sqrt{\frac{1}{K-1}\sum \limits_{k=1}^K{\left({C}_{r,k}-\overline{C_r}\right)}^2}}{\ \overline{C_r}} $$

4 $$ {IR}_{r,k}=\frac{\sqrt{\frac{1}{I-1}\sum \limits_{i\epsilon {ROI}_{r,k}}{\left({C}_{i,r}-{C}_{r,k}\right)}^2}}{C_{r,k}} $$

*K* is the number of regions (60 for each ROI size), r indicates the region size for the six different sizes used, *C*_*r*, *k*_ the average radioactivity concentration for ROI *k*, $\overline{C_r}$ the average background radioactivity concentration for all ROIs of the same size, I the number of voxels in each ROI, and *C*_*i*, *r*_ the radioactivity concentration of voxel *i* in region *r*.

In order to investigate and quantify the presence of edge artefacts, for each sphere, the axial slice with the largest diameter was selected and the surface areas of the spheres were plotted. Six 1D profiles were also drawn for each sphere on the same slice used for the surface plots, and the Peak-To-Valley (PTV) ratio was estimated with peak being the highest value along the profile and the valley the lowest within the defined regions [30].

### Clinical data

Fourteen patients aged between 51 and 79 years old with prostate cancer were injected with 163 ± 23 MBq (mean ± standard deviation) ^68^Ga-PSMA and underwent whole body PET-CT scanning approximately 1 h later, on the GE Discovery 710 scanner at the PET Centre, St Thomas’ Hospital, London, UK, between 2017 and 2018. The data were reconstructed with OSEM+TOF and PSF both smoothed with a 6.4 mm Gaussian filter, and BPL with β = 200, 400, 600, 800, 1000 and 1200.

### Analysis of clinical data

Thirteen prostate, nine lymph nodes and two bone lesions (24 lesions in total) were manually delineated on MIM Software Inc. 6.9.3 and checked by an experienced nuclear medicine physician. The log transformed mean, maximum and peak standard uptake values (SUV_mean_, SUV_max_ and SUV_peak_) were calculated, and Bland-Altman plots were generated to assess the agreement between PSF and BPL reconstructions [31].

### Statistical analyses

Welch’s t test at p<0.05 was used to compare the CRC between Fluorine-18 and Gallium-68 for the phantom data and paired t test at p<0.05 to compare the SUV values as measured from the different reconstructions for the clinical data.

## Results

### Optimisation of advanced reconstruction methods for Gallium-68

#### Edge artefacts

**Fig. 1:**
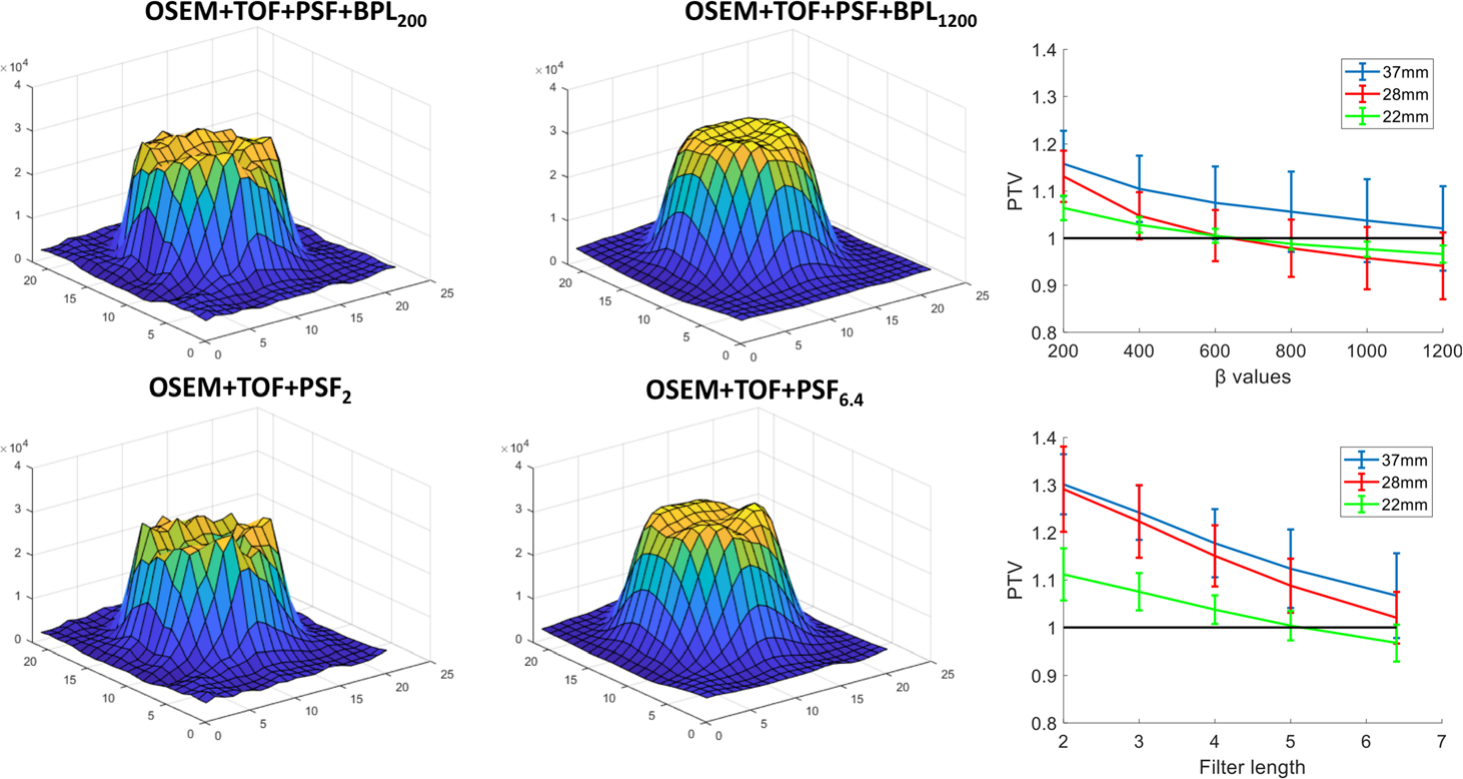
Surface plots for the 37-mm sphere of the NEMA IQ phantom taken from the slice with the largest diameter. The top row refers to BPL reconstructions with increasing penalisation factor (left to right) and the bottom row to PSF reconstructions with increasing width (left to right) of Gaussian post-reconstruction filter. The averaged peak-to-valley ratios from six profiles are also shown on the right for BPL (top) and PSF (bottom) reconstructions for the 37-, 28-, and 22-mm spheres. The error bars represent the standard deviations.

Edge artefacts were observed in both PSF and BPL reconstructions for low levels of smoothing as shown in Fig. 1. These were mainly prominent in the 37-, 28-, and 22-mm spheres while PTV could not be estimated for the rest as the surface had a “conical” rather than a “concave” shape and a PTV could not be measured. Moreover, the limited spatial resolution did not allow the drawing of more than 2–4 different profiles. For all profiles, and in the three largest spheres, the valley was identified in the centre of the region and the peak at the edges. PTV was decreased with the increase of β and Gaussian filter width. The optimum β was region dependent with 22- and 28-mm spheres requiring a value between 600 and 800 in order to achieve PTV = 1 ± 0.02 while β=1200 was required for the 37-mm sphere. Similarly, a 6.4-mm filter was required for PSF reconstruction for the 37- and 28-mm spheres to achieve PTV equal to 1.7 and 1.02, respectively, and 5-mm for the 22-mm sphere to achieve PTV=1.

#### Recovery coefficients

**Fig. 2:**
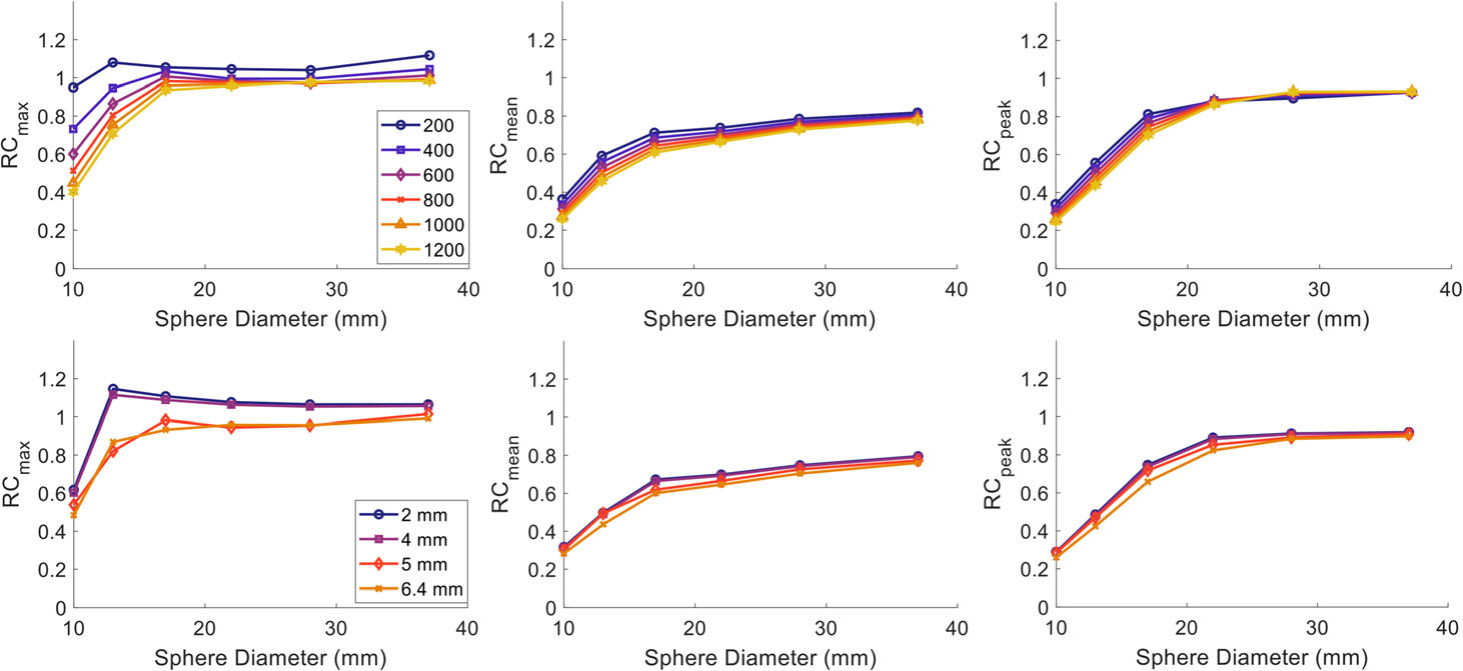
RC_max_ (left), RC_mean_ (middle) and RC_peak_ (right) for Gallium-68 measured from the NEMA IQ phantom scanned on a GE Discovery 710 scanner and reconstructed using BPL with varying penalisation factor (top row) and PSF with varying post-reconstruction Gaussian filter width (bottom row).

RCs decreased with increasing Gaussian filter for PSF as shown in Fig. 2. For RC_max_, minimal effect was observed for filters up to 4-mm wide (less than 2.9% change for any of the spheres). When a 5-mm filter was applied though, the decrease in RC_max_ ranged between 4 and 26%. A more conservative decrease with wider smoothing filter was noticed in RC_mean_ and RC_peak_ where a maximum of 12% decrease was noticed between the 2 and 6.4 mm for both RC_mean_ and RC_peak_. The largest differences for all recovery metrics were noticed for the 13-mm sphere which exhibited an overshoot in RC_max_, i.e. a sudden increase in the curves with recoveries >1, for filter widths smaller than 5 mm.

A similar pattern was observed for the BPL reconstruction with increasing penalisation factor. The RC_peak_ was the least sensitive metric for regions larger than 22 mm for which, changes smaller than 5% between BPL_200_ and BPL_1200_ were observed. RC_max_ did not change by more than 3.5% for spheres >17 mm when increasing β to values higher than 600 while differences for the smallest spheres were 4 times higher and the curves became smoother.

#### Recovery coefficient for Gallium-68—Standard iterative reconstruction

**Fig. 3:**
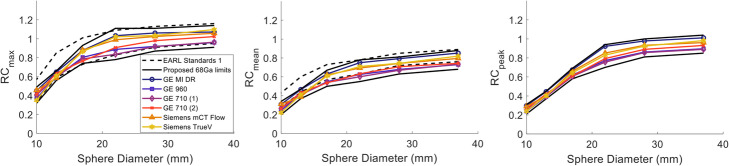
RC_max_ (left), RC_mean_ (middle) and RC_peak_ (right) for Gallium-68 from all six centres measured from the NEMA IQ phantom reconstructed with standard OSEM. The RCs (dashed black lines) for the EARL 1 FLuorine-18 standards are also displayed as opposed to the limits proposed in this study for Gallium-68 (black solid lines)

**Fig. 4:**
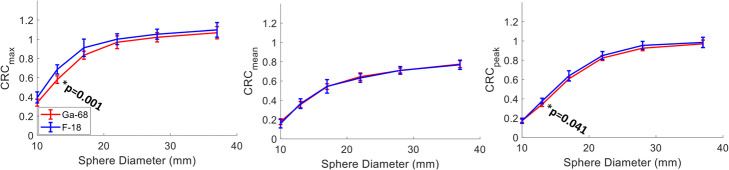
Average CRC_max_ (left), CRC_mean_ (middle) and CRC_peak_ (right) for all 6 Gallium-68 (blue curve) and the 12 Fluorine-18 (red curve) acquisitions reconstructed with standard OSEM. The error bars represent the standard deviation between sites. Significant difference was noticed for the 13-mm sphere at the 5% significance level for CRC_max_ and CRC_peak_

The Gallium-68 RC curves for each of the six sites along with the EARL standard 1 and the comparison of the average CRC with Fluorine-18 are shown in Figs. 3 and 4, respectively. The RC_mean_, RC_max_ and RC_peak_ for Gallium-68 were at the lower limit of the EARL specifications, especially for the three smallest spheres. The RC_means_ for the four largest spheres for three sites were between 1 and 7% lower than the lower EARL limit specified for Fluorine-18, while the 10- and 13-mm spheres were between 1 and 19% lower for five out of six sites. RC_max_ was within limits for all sites.

No significant difference was found between the average CRC_mean_ of all six Gallium-68 scans and the 12 Fluorine-18 scans (all of which were within the EARL specifications) as shown in Fig. 4. CRC_max_ and CRC_peak_ were comparable for the three largest spheres but consistently lower for Gallium-68. Welch’s t test indicated statistically significant differences between Gallium-68 and Fluorine-18 for CRC_max_ and the CRC_peak_ for the 13-mm sphere.

### Standardisation of BPL and PSF reconstruction for Gallium-68

#### Investigation of recovery coefficients

**Fig. 5:**
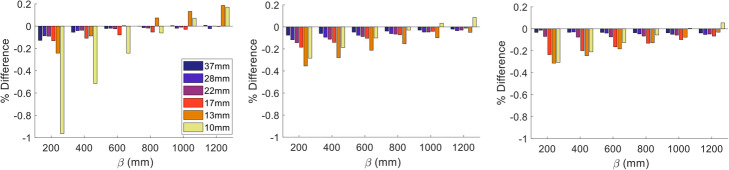
Percentage difference of RC_max_ (left), RC_mean_ (middle) and RC_peak_ (right) between BPL_β_ and PSF_6.4_ reconstruction [(${RC}_{BPL_{\beta }}-{RC}_{PSF_{6.4}}\left)/{RC}_{PSF_{6.4}}\right]$ for all spheres. Each group of bars corresponds to the comparison with a single β value (200, 400, 600, 800, 1000 and 1200)

Since all scanners have the ability to perform PSF reconstructions while only the GE scanners have the BPL functionality, BPL was standardised to PSF. Therefore, the various BPL_β_ reconstructions from GE 710 (1) were compared against PSF_6.4_ from the same scanner. As shown in Fig. 5, BPL_800_ was the reconstruction which closely agreed to PSF_6.4_ for the RC_max_ with a maximum of 8% difference being observed for the 13-mm sphere. Higher penalisation factors seemed to produce lower maximum values for the BPL compared to PSF_6.4_ reconstruction, especially for the two smallest spheres. Similarly, RC_mean_ and RC_peak_ agreement were region-related with BPL_1200_ having the best agreement (<5%) for all but the smallest sphere where differences of 10% and 7% were observed for RC_mean_ and RC_peak_, respectively. BPL_800_ exhibited the best agreement for the 10-mm sphere (1.7%) with the differences in the rest ranging between 2.6 and 13.8% for RC_mean_ and 2.4 and 11.9% for RC_peak_.

**Fig. 6:**
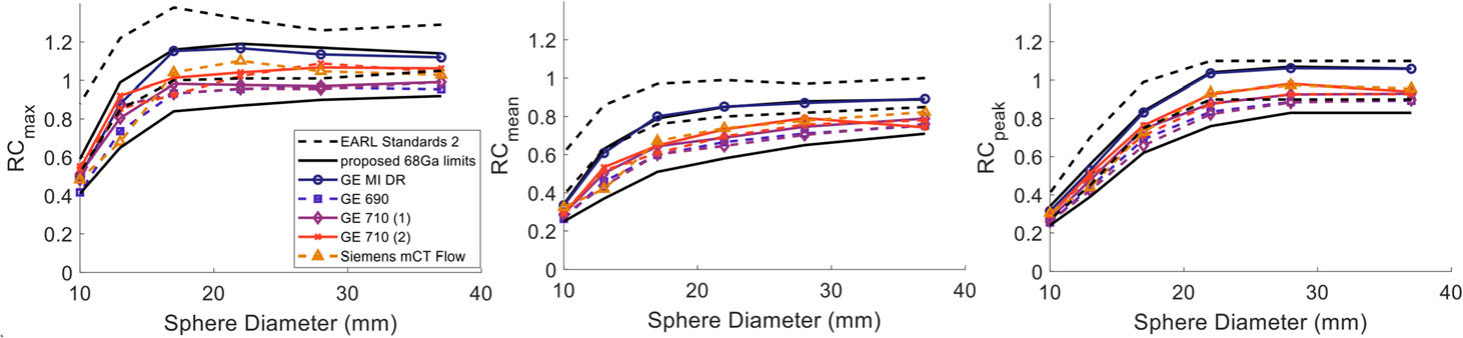
RC_max_ (left), RC_mean_ (middle) and RC_peak_ (right) for Gallium-68 from all five centres measured from the NEMA IQ phantom reconstructed with BPL_800_ (solid lines) and PSF_6.4_ for the GE scanners and PSF_5_ for the Siemens scanner (dashed lines). The 2 RCs (dashed black lines) for the EARL 2 Fluorine-18 standards are also displayed as opposed to the limits proposed in this study for Gallium-68 (black solid lines)

The RCs in Fig. 6 did not show any systematic or noticeable differences between BPL_800_ and PSF_5/6.4_ (PSF_5_ and PSF_6.4_) reconstructions. The BPL_800_ from the GE MI DR scanner provided higher RC values compared to the rest, with differences of up to 21% for RC_max_ and RC_mean_ and up to 29% for RC_mean_ when compared to the PSF_6.4_ from the GE 690 which in general provided the lower values. Nevertheless, the ranges of the RC were comparable to the width of the EARL Fluorine-18 standard 1 and 2 specifications, depending on sphere size, with the later defined for advanced reconstruction methods.

**Fig. 7:**
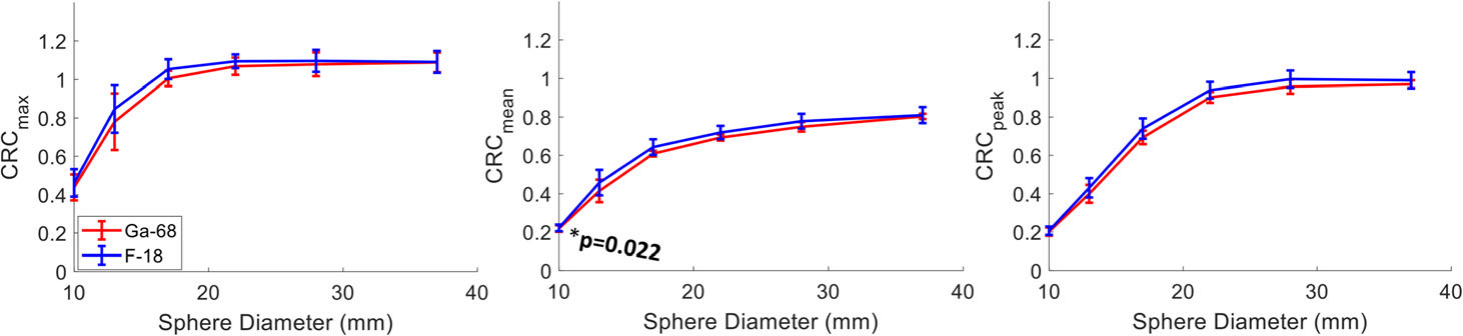
Average CRC_max_ (left), CRC_mean_ (middle) and CRC_peak_ (right) for all 6 Gallium-68 (blue curve) and the 12 Fluorine-18 (red curve) acquisitions. All Fluorine-18 acquisitions have been reconstructed with PSF_6.4/5_, and the Gallium-68 includes all PSF_6.4/5_ and BPL_800_ reconstructions. The error bars represent the standard deviation between sites. Significant difference was noticed for the 10-mm sphere at the 5% significance level for CRC_mean_

The average CRC values from all advanced reconstruction methods for Gallium-68 were also comparable to the ones for Fluorine-18 averaged from 12 different sites with the images reconstructed using PSF_5/6.4_ (Fig. 7). A significant difference was found for CRC_mean_ in the 10-mm sphere.

#### Investigation of noise characteristics

**Fig. 8:**
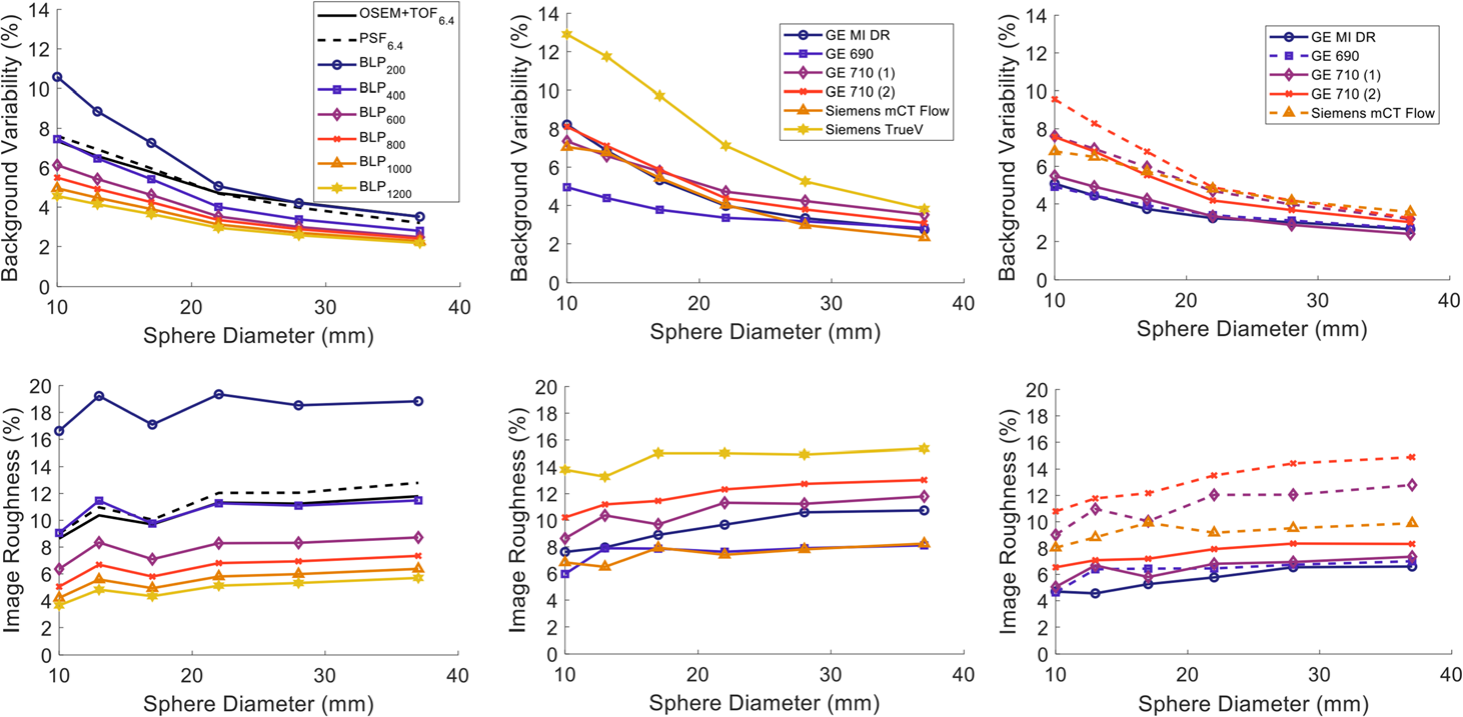
Background variability (top row) and image roughness (bottom row) for Gallium-68 acquisitions of the NEMA IQ phantom. The left column displays the metrics for varying β values of the BPL reconstruction including OSEM+TOF_6.4_ and PSF_6.4_, the middle column the OSEM and OSEM+TOF reconstructions for six different sites and the right figure for the advanced reconstructions methods

Both BV and IR were at similar levels for both standard OSEM+TOF and PSF when smoothed with a 6.4 mm Gaussian filter as shown in Fig. 8. Both noise metrics decreased with increasing β for BPL. A β value of >400 was required in order for the images to have a similar or lower noise level compared to OSEM+TOF and PSF with BV ranging between 7.5 and 2.8% and IR between 9.1 and 11.5%. A sharp decrease of 31.6–48.1% and 61.0–69.6%, depending on the region size, was observed for BV and IR, respectively, when using BPL_800_ compared to BPL_200_ while additional increase in β resulted in more moderate improvements.

The images generated with 2D reconstruction from the Siemens TrueV exhibited the highest values (~15% IR and 13% BV for the small sphere) as illustrated in the middle row of Fig. 8. For the rest of the sites BV did not exceed 8% and IR 13% for any of the spheres when reconstructed with standard OSEM+TOF. Comparable noise levels were measured for the PSF_6.4_ reconstructions while BPL_800_ exhibited ~40% lower IR and between 6.6 and 27.8% lower BV (depending on the region size) compared to their corresponding PSF reconstructions.

### Definition of standardisation specifications

The proposed limits for Gallium-68 trials for both OSEM and advanced reconstruction methods were defined by taking ±2 standard deviations from the mean RCs of all submitted images [32] and are shown in Table 2. The proposed limits for RC_max_ were comparable with the ones proposed by EARL Fluorine-18 standard 1 for the 4 largest spheres with the ones proposed in this study being up to 8% lower. For the 13-mm and 10-mm spheres though, the upper limit of RC_max_ was 23% and 14% lower, respectively. The proposed limits for RC_mean_ were up to 12% lower for the four largest spheres when compared to EARL Fluorine-18 standard 1 while for the two smaller spheres, up to 27% lower.

**Table 2:** Proposed specification limits for Gallium-68 multi-centre trials

Sphere diameter (mm)	Conventional reconstruction (OSEM_**5/6.4**_, OSEM+TOF_**5/6.4**_)			Advanced reconstruction (PSF_**5/6.4**_, PSF_**800**_)		
	SUV_**max**_	SUV_**mean**_	SUV_**peak**_	SUV_**max**_	SUV_**mean**_	SUV_**peak**_
**37**	0.91–1.14	0.68–0.88	0.85–1.04	0.92–1.14	0.71–0.89	0.83–1.06
**28**	0.87–1.11	0.63–0.81	0.81–1.00	0.90–1.17	0.65–0.88	0.83–1.07
**22**	0.78–1.11	0.55–0.78	0.70–0.94	0.87–1.19	0.58–0.85	0.76–1.04
**17**	0.74–0.93	0.50–0.67	0.58–0.69	0.84–1.16	0.51–0.79	0.62–0.84
**13**	0.56–0.66	0.37–0.47	0.37–0.45	0.65–0.99	0.37–0.63	0.39–0.56
**10**	0.32–0.49	0.20–0.34	0.21–0.31	0.41–0.59	0.25–0.34	0.24–0.34

Similarly, when compared to EARL Fluorine-18 standard 2, both the upper and lower proposed RC_max_ limits for advanced reconstruction methods, were up to 16% lower for the four largest spheres but for the two smallest spheres were between 19 and 33% lower. Lower limits are also proposed here for RC_mean_, with the decrease ranging between 11 and 25% for the lower and 16–41% for the upper limit compared to EARL Fluorine-18 standard 2. When compared to the proposed limits for conventional OSEM reconstruction, the three largest spheres had comparable RC_max_ and RC_mean_ specifications but considerably higher for the rest with RC_max_ being 13–28% and 19–51% higher and RC_mean_ 19–33 and 3–27% higher for the upper and lower limit, respectively. RC_peak_ was also 22% and 24% higher for spheres 17 and 13 mm when an advanced reconstruction was performed but less than 11% for the rest of the limits.

### Assessment of clinical data

**Fig. 9:**
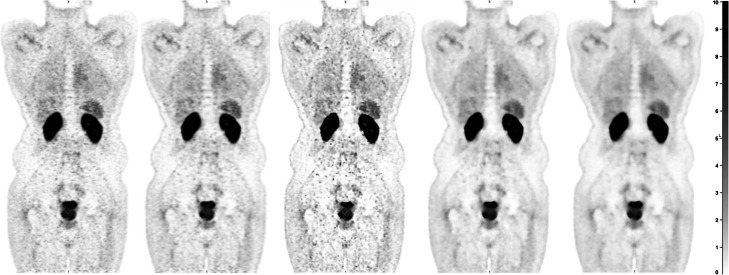
Coronal views of prostate cancer patient, scanned with ^68^Ga-PSMA with the data reconstructed using OSEM+TOF_6.4_, PSF_6.4_, BPL_200_, BPL_800_ and BPL_1200_ from left to right

Consistent with the phantom data, visual inspection of the reconstructed clinical images, indicated reduced noise for images reconstructed with BPL_800_ compared to the rest of the images (Fig. 9). Minimal differences in terms of noise but reduced contrast were observed with increasing β.

**Fig. 10:**
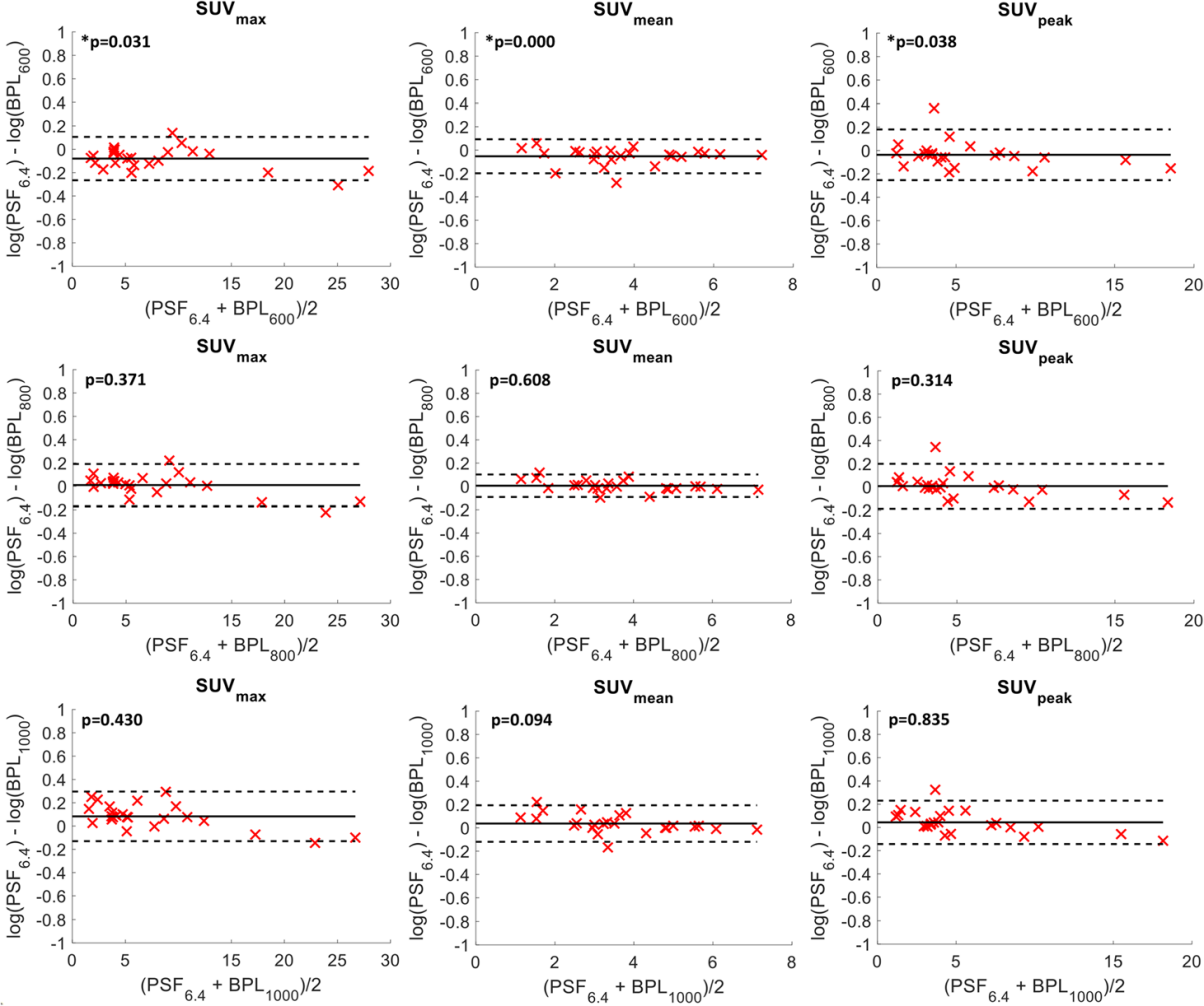
Bland-Altman plots for SUV_max_ (left column), SUV_mean_ (middle column) and SUV_peak_ (right column) of all 22 lesions with the black solid line being the average difference of the log-transformed SUVs between PSF_6.4_ and BPL_600_ (first row), BPL_800_ (middle row) and BPL_1000_ (bottom row) and the dashed lines 1.96 x the standard deviation. The p value from the paired t test is also quoted on each figure with * indicating significant differences at the 5% significance level

The Bland-Altman plots in Fig. 10 indicated that all but one lesion for SUV_peak_ were within the 95% confidence intervals when comparing SUVs between PSF_6.4_ and BPL. Similar results were observed for SUV_max_ and SUV_mean_ with a maximum of 2 lesions being outside the 95% confidence intervals. The only BPL reconstructions for which the SUVs were not significantly different from PSF_6.4_ were BPL_800_ and BPL_1000_. When comparing PSF_6.4_ to OSEM+TOF_6.4_, the difference in SUV_max_ ranged between −19 to 29%. SUV_mean_ and SUV_peak_ were more comparable between reconstructions with PSF_6.4_ being up to 14% higher.

## Discussion

In this study, it was shown that multi-centre trials using Gallium-68 based tracers are feasible for both conventional iterative and advanced reconstruction methods which include PSF with or without a Bayesian penalisation likelihood algorithm (Q.CLEAR) by adhering to the adjusted specifications. Those specifications mainly differ from the ones defined by EARL Fluorine-18 standards 1 and 2 for the smallest spheres of the NEMA IQ phantom. Still, two different sets of limits were needed for conventional and advanced reconstructions, similar to EARL for Fluorine-18, as the two methods cannot be used interchangeably. A PSF reconstruction using a Gaussian post-reconstruction filter of approximately 6 mm and an BPL reconstruction with penalisation value of 800–1000 were needed for the two methods to provide comparable results, suppress the edge artefacts and for BPL to provide images with lower noise levels when compared to PSF. The results from the phantom study regarding the agreement of the two advanced reconstruction methods in terms of SUVs were also in agreement with the results from clinical analysis.

Consistent with the literature, the RCs for Gallium-68 were at the lower limits of the EARL Fluorine-18 standard 1 for conventional iterative reconstruction [11, 33–35]. No additional correction factors were needed in this study to account for well counter variability [11] as they all had traceability to the National Physics Laboratory. The calibration factor used for Gallium-68 was therefore locally defined if it was deemed necessary rather than using the one specified from the manufacturer. Moreover, all scanners had to provide 2-week results from their daily quality assurance scans as part of their accreditation procedure for the UK PET Core Lab (http://www.ncri-pet.org.uk/) to assess their stability and accuracy. Therefore, no additional correction for system calibration bias was needed either [32]. The observed decrease was related to blurring due to the higher positron range of Gallium-68 when compared to Fluorine-18 and was, as expected, mainly apparent in the smaller spheres of the NEMA IQ phantom [13]. In the corresponding comparison when PSF was included, there seemed to be an even better agreement between the CRCs of the two isotopes. This could be attributed to the fact that blurring due to positron range was partially accounted for.

The proposed specification limits in Table 2 depict the aforementioned results with the limits for the OSEM reconstruction being similar to the ones proposed by EARL Fluorine-18 standard 1 for the four larger spheres and lower for the smaller spheres in which the blurring due to positron range is more prominent. Despite the consensus of decreased recovery in Gallium-68 images, it has been proposed that the EARL specifications can still be applied as the curves are not noticeably different [11, 33]. Although this approach might be feasible, it could be difficult to manage as Gallium-68-based multi-centre trials become more popular and a large number of perfectly functional scanners would be prohibited from participating. For example, in Fig. 3, five out of six scanners have at least one sphere with RC below the lower limit of EARL Fluorine-18 standard 1. Similarly, in the multi-centre study performed by Huizing et al. [11], at least 5 out of 13 scanners did not meet the EARL criteria, even after the proposed correction for variances in the well counter, while valid results presented in other studies would also render the scanners ineligible from participating in multi-centre trials [33, 35]. The proposed limits in this study are closer to previously published results [11, 33–35] and consistent with the observation by Huizing et al. of higher variation between PET-CT systems [11]. Despite the fact that all scanners performed similarly in this study in terms of recovery, it needs to be taken into account that only six were used for the definition of the specification limits which is a limitation of this study, including scanners of different generations such as the Siemens Biograph TrueV and the GE MI DR. Since according to the EARL specifications the results from the TrueV scanner would be acceptable for participation in multi-centre trials with a number of those scanners still operational and used in research, it was decided to include it in this study but highlight the effect of the noise in comparison to the rest of the scanners. Moreover, this scanner complies with the EARL standard 1 which includes scanner without TOF capability. In addition, no Philips scanners participated in this study although the limits proposed in here are expected to be applicable for those scanners as well based on previous studies which did not notice any systematic differences between manufacturers [11, 32]. Nevertheless, it might be useful to revisit those limits in the future when most of the old scanners will be decommissioned and to evaluate them in studies were Philips scanners also participate.

A potential limitation when comparing the CRCs between Gallium-68 and Fluorine-18 scans is the difference in the sphere-to-background ratio between the different acquisitions. To investigate this effect, the NEMA IQ phantom was scanned on the GE-710 (1) scanner with sphere-to-background ratios ranging from 2:1 to 15:1, reconstructed using OSEM with time-of-flight and smoothed with a post-reconstruction Gaussian filter of 6.4 mm. The results displayed in Suppl. Figure 1 indicated that all coefficients were comparable for ratios between 5:1 and 10:1. Partial volume effects became more prominent for smaller ratios for all spheres while for larger ratios, the smaller sphere was more accurately measured. The range of ratios for the phantoms included in this study though was within the acceptable limits. Moreover, the level of the post-reconstruction smoothing is expected to have suppressed any edge artefacts within this range according to the results of this study, even in the case of PSF reconstruction.

Another point of consideration in this study is the difference in the reconstruction parameters especially in terms of voxel size between scanners. The recovery curves however were comparable and within the variation expected due to reproducibility between the images from scanner with the largest voxel size (GE Discovery 690) and the scanner with the smallest size (GE Discovery 710) for both conventional and advanced reconstructions. As expected, the effect of the largest voxel is highlighted in the lower noise for that acquisition. Similar results have also been reported in the literature where images reconstructed with a comparable range of voxels sizes have been included [11, 32].

As expected, edge artefacts were apparent in both PSF and BPL reconstructions which were mitigated with increased Gaussian filtering and β value, respectively. Consistent with the literature, the characteristic increase of the RC_max_ in the 13-mm sphere was noticed in both reconstructions due to the superimposition of the edges for this region which are heavily influenced by the edge artefacts [14, 17, 32]. For the 10-mm sphere, this effect is countered by the partial volume effects. Assessment of the recovery curves on their own would have made it difficult to evaluate the level of suppression of the edge artefacts as only the 13-mm sphere directly indicated the problem in the images. The surface curves demonstrated the level each region was affected and their respective smoothing requirements. This could be useful for a study interested in protocol optimisation when the approximate dimensions of the region of interest are known. Moreover, in studies where metrics such as the SUV_max_ and SUV_peak_ are applied, it is important for a similar assessment to be followed in order to avoid usage of voxels affected by the artefacts.

The required level of Gaussian filtering necessary to partially correct the edge artefacts in PSF reconstruction, also resulted in images with similar noise levels in terms of image roughness and background variability as the standard OSEM+TOF reconstruction when smoothed with the same filter, in accordance to the results from Tong et al. [25]. Even though a β value of 400 also resulted in images with similar noise properties, it did not seem sufficient for addressing the edge artefacts and a higher value was required which resulted in even lower noise levels but at the expense of recovery.

The optimum β value needed for standardising the recovery of BPL with the PSF reconstruction was region-dependant but the best agreement on average was observed for β=800. For larger β values, the recovery from the two smaller spheres, which were the ones mainly benefited from PSF and BPL reconstructions, was approximately 20% lower compared to PSF. This was in agreement with the clinical results for which all BPL reconstructions except BPL_800_ and BPL_1000_ were found to be significantly different compared to PSF_6.4_. Moreover, visual assessment of the images indicated minimal effects in image quality when increasing β to values higher than 800. Therefore, BPL_800_ is proposed for standardising Gallium-68 based PET acquisitions reconstructed with PSF and BPL.

The focus of this study was to harmonise the two types of reconstruction rather than optimising them. Depending on the tumour size, it could be argued that for a single-centre study focused on BPL reconstruction a slightly smaller β value such as 600 would provide higher contrast with acceptable noise levels. In such an approach, the assessment of the noise characteristics would need to be part of the optimisation process. In addition, better results both in terms of accuracy and noise could be expected if an isotope-specific PSF was implemented [36]. For this study, the data were reconstructed on the scanner though, using the manufacturer’s PSF as that would be the case for any prospective clinical trial. Ideally, taking into account the considerable difference in positron range between Fluorine-18 and Gallium-68, the advanced reconstruction methods would have been reconstructed with a Gallium-68specific PSF. Nonetheless, the selected level of Gaussian smoothing and penalisation value for standardising the PSF and BPL reconstruction, retained the improved recovery, especially in the smaller regions, and lower noise when compared to OSEM+TOF while they were found to be the minimum values for minimising the edge artefacts.

This study also indicates that isotope-specific limits might be necessary for performing multi-centre trials especially for isotopes with distinctly different positron ranges. As more and more isotopes find their way into the clinic though, this might prove logistically difficult as it could result in multiple standards that need to be regularly reviewed and updated. A method to move this field forward might be to establish a model for defining the limits based on the isotopes’ characteristics [37]. An alternative approach could be to utilise newly developed deep learning approaches to optimise the images [38]. The latter could potentially convert the images from each scanner to the samestandard and improve the quality in a more automated process, allowing direct image comparison between scanners. For the time being though, where the number of isotopes used in multi-centre trials is limited, careful assessment of the specification limits is needed.

## Conclusion

Consistent with the literature, the recovery coefficients of Gallium-68 PET-CT performed near and below the lower levels of the EARL1 performance specifications for Fluorine-18 and well below the recently defined levels for the smaller spheres in the NEMA IQ phantom. Therefore, new Gallium-68 specific levels are proposed for multi-centre trials. Gaussian filtering of 6 mm (or more) and a penalisation value of 800 (or more) were required to minimise edge artefacts from the majority of the regions from the phantom in reconstructions using PSF and PSF with Bayesian penalisation likelihood, respectively. Those two reconstructions also provided similar recovery coefficient results for the phantom and similar SUVs in a clinical assessment of patients with prostate cancer. Moreover, they resulted in similar or lower noise levels and higher recovery coefficients when compared to standard iterative reconstruction and separate performance specifications for advanced reconstruction methods were defined.

## Supplementary Information

**Additional file 1: Suppl. Figure 1.** Effect of different sphere-to-Background ratios. CRC_max_ (left), CRC_mean_ (middle), CRC_peak_ (right) for five PET-CT acquisitions of the NEMA IQ phantom with varying sphere-to-background ratio. For a ratio of 2:1 only the two largest spheres could be defined using the 3D isocontour at 50% of the maximum.

## Data Availability

The datasets used and analysed during the current study are available from the corresponding author on reasonable request.
