# Supplementary figures and images for: Standardisation of conventional and advanced iterative reconstruction methods for Gallium-68 multi-centre PET-CT trials
Source: EJNMMI Phys. 2021 Jul 17;8:52. doi: 10.1186/s40658-021-00400-8 (PMC8286213; doi:10.1186/s40658-021-00400-8)

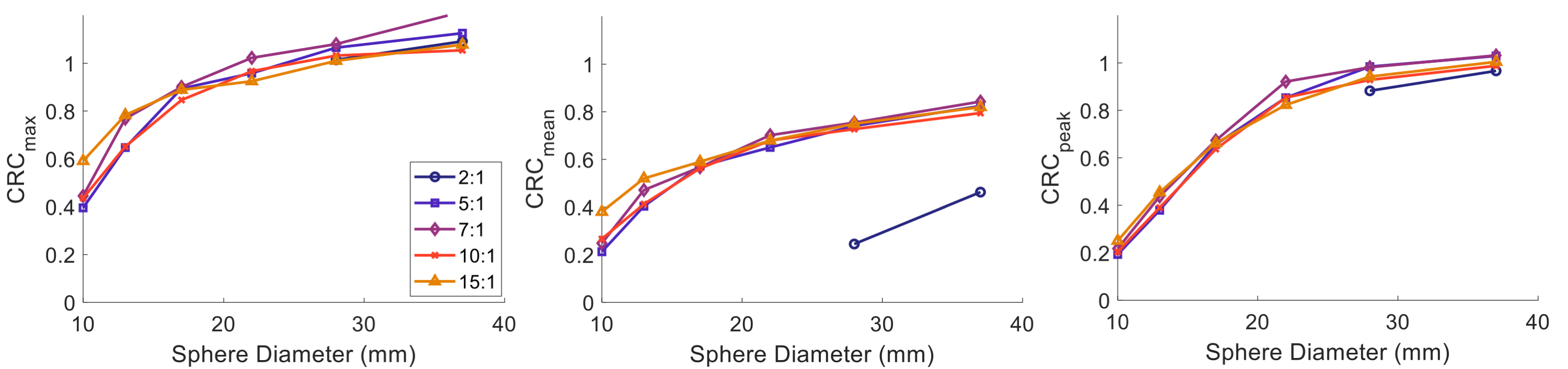

Supplement: Supplementary file 1 — Additional file 1: Suppl. Figure 1. Effect of different sphere-to-Background ratios. CRCmax (left), CRCmean (middle), CRCpeak (right) for five PET-CT acquisitions of the NEMA IQ phantom with varying sphere-to-background ratio. For a ratio of 2:1 only the two largest spheres could be defined using the 3D isocontour at 50% of the maximum. [file 40658_2021_400_MOESM1_ESM.tiff]
